# The Monkey’s Paw^1^

**DOI:** 10.3201/eid1709.AC1709

**Published:** 2011-09

**Authors:** Polyxeni Potter

**Affiliations:** Author affiliation: Centers for Disease Control and Prevention, Atlanta, Georgia, USA

**Keywords:** art science connection, emerging infectious diseases, art and medicine, Frans Snyders, the monkey’s paw, Flemish painting, animaliers, still life with animals, influenza

**Figure:**
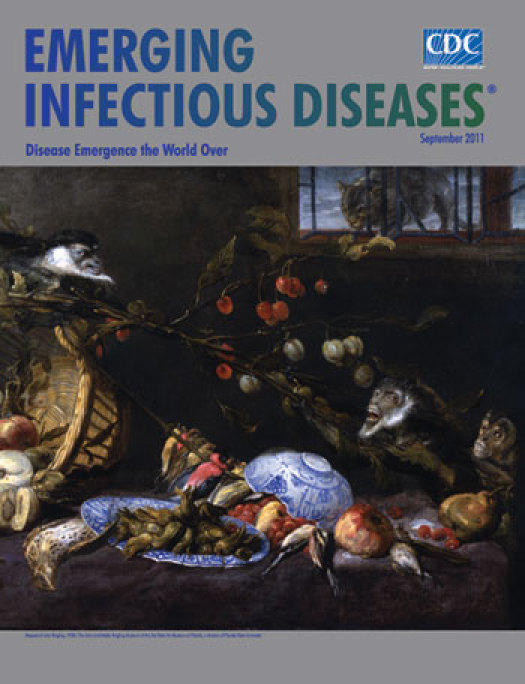
**Frans Snyders (1579–1657). *Still Life with Fighting Monkeys* (1635) Oil on canvas (74.9 cm × 108 cm).** Bequest of John Ringling, 1936, The John and Mable Ringling Museum of Art, the State Art Museum of Florida, a division of Florida State University

Unexpected consequences fuel the creative mind. The stuff of adventure in literature, their twists and turns wreak havoc in the science laboratory and the art studio alike. Frans Snyders, old master and founder of Baroque still life with animals, understood the unexpected and its power to surprise and used it to animate his work. A thriving arts market in 17^th^-century Flanders, supported by growing prosperity and curiosity about the natural world, provided a rich environment for still life painting, known since antiquity but always relegated low down the ladder of genres.

As he expressed the exuberance of his age in lifeless objects made dynamic and relevant, Snyders elevated the genre. His compositions often contained live animals as well as carcasses at the butcher shop or game fresh from the hunt. He so loved this part of the work that he abandoned still lifes altogether to become one of the first animaliers. He collected specimens of local and exotic animals to observe their behavior and physical characteristics and improve his specialized portrayal of them.

Snyders was born in Antwerp, center of the arts during the Counter Reformation and playground of such luminaries as the Brueghel family of painters, Peter Paul Rubens, and Anthony van Dyck. A student of Pieter Brueghel the Younger, Snyders managed to convey the local culture not just with accuracy but with humor and commentary in compositions later considered best of the genre. He joined the Antwerp painters Guild of St. Luke and, like other serious artists of his time, visited Italy to study the masters. He was competent and prolific and attracted royal patronage and great popularity at home and abroad.

He enjoyed the friendship of the best artists of his day rather than being overshadowed by them. Rubens, whose star eclipsed all those around him, admired his skills and commissioned him to paint animals and still life elements in many of his works. Once, when a patron could not tell their work apart, Rubens consented that no one could depict dead animals better than Snyders, though when it came to live ones, he, Rubens, was the best. Van Dyck painted several portraits of Snyders, who was related to the Connelis de Vos family of painters by marriage and counted among his students and associates Jan Fyt, a still life master in his own right.

Animal still life—hunting or market scenes, butcher stalls, kitchen pantries―were transformed from static displays to vibrant collections of specimens shown to best advantage, colored with symbolism, and injected with humanity and interest. In his more than 50-year career, Snyders developed and refined his skills, leaving behind many paintings and drawings, hundreds of which survive. *Still Life with Fighting Monkeys*, on this month’s cover, contains many of the artist’s finest features.

Middle-class folks were not allowed to hunt in Snyders’ Flanders, only the nobility. And though this painting does not show large trophy game, the row of colorful finches secured on a willow branch and small birds lying pathetically on the edge of the table hint at the status of this household―also home of such exotic pets as the mischievous monkeys in the center of the action. In the artist’s style, what might have been a sedate tabletop scene is enlivened by altercating primates, themselves joined by hostile feline intruders.

Having toppled the basket, upsetting the fruit and scattering the arrangement, the monkeys tugged nervously on the branch of rainier cherries already manhandled and jutting off to nowhere. The finch display collapsed in a heap, china overturned and worse, and two angry cats ready to pounce from opposite ends complete the picture. Snyders’ skills shine in the fur of the live animals and the texture of the game birds, which far from rigid or damaged by the hunt are soft and languid as they rest on their backs human-like. The fruit is plump and enticing, even rolled to the edge of the cloth. Small branches with crinkly leaves add to the natural feel of the original arrangement.

Monkeys were frequent visitors in Flemish paintings of this era, often linked to excess and greed, their troublesome anthropomorphic features mimicking the foolish aspects of human behavior. Shameless and unruly, they invite symbolism in this scene: the best choreographed arrangements could be instantly ruined by the slightest intrusion. This not only in still life painting but anywhere the law of unintended consequences applies, and no less in public health, where each day nature’s basket is toppled by unexpected ecologic, social, and biologic paws.

In this issue of Emerging Infectious Diseases alone, diverse offerings from around the world attest to the immense influence of the monkey’s paw, particularly when another creature inflames an already dangerous situation. Such is the case with influenza. In the past century, three pandemics swept the globe in which viruses from birds likely played a role. A new strain, influenza A virus (H5N1), spread through bird populations across Asia, Africa, and Europe, infecting domesticated birds, including ducks and chickens, and long-range migratory birds. Its first recorded appearance in humans was in Hong Kong in 1997.

Each time a new element of uncertainty is thrown into the mix, what will come out and how it will behave become more difficult to predict. Snyders knew this when he painted the fighting monkeys and the cats in his still life in Flanders. But the monkey’s paw in pandemic influenza remains to be seen.
